# Molecular changes in endometrium origin stromal cells during initiation of cardiomyogenic differentiation induced with Decitabine, Angiotensin II and TGF- β1

**DOI:** 10.1038/s41598-024-68108-0

**Published:** 2024-07-23

**Authors:** Giedrė Skliutė, Giedrė Staponkutė, Edvinas Skliutas, Mangirdas Malinauskas, Rūta Navakauskienė

**Affiliations:** 1 Department of Molecular Cell Biology, Life Sciences Center, Institute of Biochemistry, Vilnius University, Saulėtekio Av. 7, 10257 Vilnius, Lithuania; 2 Faculty of Physics, Laser Research Center, Vilnius University, Saulėtekio Av. 10, 10223 Vilnius, Lithuania

**Keywords:** Cardiomyogenic differentiation, Endometrial stromal cells, Endometriosis, Menstrual stromal cells, Cell biology, Molecular biology, Stem cells

## Abstract

Stem cells’ differentiation toward cardiac lineage is a complex process dependent on various alterations in molecular basis and regulation pathways. The aim of the study is to show that endometrium-derived stromal cells – menstrual, endometrial and endometriotic, could be an attractive source for examination of the mechanisms underlying cardiomyogenesis. After treatment with Decitabine, Angiotensin II and TGF-β1, cells demonstrated morphological dedifferentiation into early cardiomyocyte-like cells and expressed CD36, CD106, CD172a typically used to sort for human pluripotent stem cell-derived cardiomyocytes. RT-qPCR revealed changed cells’ genetic profiles, as majority of cardiac lineage differentiation related genes and cardiac ion channels (calcium, sodium, potassium) coding genes were upregulated after 6 and 13 days of exposure. Additionally, analysis of expression of various signaling proteins (*FOXO1, PDGFB, TGFBR1, mTOR, VEGFA, WNT4, Notch1*) coding genes showed differences between cell cultures as they seem to employ distinct signaling pathways through differentiation initiation. Early stages of differentiation had biggest impact on cardiomyogenesis related proteins (Nkx-2.5, EZH2, FOXO3a, H3K9Ac) levels, as we noticed after conducting Western blot and as expected, early cardiac transcription factor Nkx-2.5 was highly expressed and localized in nucleus of differentiating cells. These findings led us to assess endometrium origin stromal cells’ potential to differentiate towards cardiomyogenic lineage and better understand the regulation of complex differentiation processes in ex vivo model systems.

## Introduction

Over the last years human pluripotent stem cells' and somatic stromal stem cells’ ability to differentiate in a cardiomyogenic lineage has been extensively investigated. The highest interest of in vitro studies mainly comes to two topics: firstly, creating a model system for researching cardiomyogenesis, cardiotoxicity and cardiac diseases, and secondly, focusing on the field of regenerative medicine^1^. However, such application perspectives remain challenging and require deep knowledge in the molecular level and regulation mechanisms of the cardiomyogenic differentiation process. Various types of stem cells have been studied to examine molecular pathways underlying cell cardiac lineage differentiation^2^. In this article, we showed that human endometrium-derived stromal cells could also be an attractive source for such a purpose. Endometrium origin stromal cells not only can be obtained from a woman’s endometrium during a biopsy procedure but also can be harvested from menstrual blood, enabling an accessible way for stromal cell isolation and cultivation^3^. Endometriosis tissue is another tissue of interest in our study, due to the importance of WNT pathways in both development of endometriosis and cardiomyogenic differentiation^4^. Versatile examination of normal and pathological cells could give reliable information on fundamentals happening through the process of cardiomyogenic differentiation of endometrium-derived cells.

One of the commonly used approaches to induce differentiation into cardiomyocyte-like cells is by influencing them with various chemical agents or biomolecules. Gasiūnienė et al*.* have successfully accomplished it before by inducing differentiation with agents Decitabine, Angiotensin II, TGF-β1 (Transforming growth factor beta 1) and others on human amniotic fluid-derived mesenchymal stem cells^5,6^, however, these agents’ effect based on regulation and signaling networks still require further investigation. Decitabine works as a hypomethylation agent. Via its action mechanism, Decitabine can lead cells into differentiation processes because of the alterations in gene expression profile^7^. Angiotensin II is the main effector molecule of the renin-angiotensin hormonal system (RAS) that is directly linked to the cardiovascular system by affecting blood pressure and vascular tone. This agent acts mainly through stimulating its own receptors that induce downstream signals for instance MAPK/ERK, Ras/Rho, or PI3/Akt pathways and by increasing expression of early growth-response genes like transforming growth factor beta (*TGFB*), platelet-derived growth factor (*PDGFB*), and vascular endothelial growth factor (*VEGF*)^8^. TGF-β1 is a member of the transforming growth factor β cytokine superfamily that is also involved in cardiovascular homeostasis and renal renin-angiotensin system modulation^9^. There are multiple signaling mechanisms of this molecule, the most widely examined is the pathway, where TGF-β1 bounds to specific receptors and induces phosphorylation of Smad proteins that results in altered transcription levels, leading to changes in gene and protein expression of differentiated cells^10^. Moreover, studies shown that TGF-β1 and Angiotensin II signals can be crosslinked, since Angiotensin II can increase the natural production of TGF-β molecules in differentiating cell^8^.

In this study, we examined three cardiomyogenic differentiation inducers’ – Decitabine, Angiotensin II and TGF-β1, effect on endometrium origin stromal cells, including menstrual (MenSCs), endometrium (EndSCs) and endometriosis (EmsSCs) stromal cells, and evaluated their ability to demonstrate cardiac-like phenotype as well as investigated the regulation processes that directed the cells to undergo these changes. Firstly, we captured alterations in cell morphology by using light and scanning electron microscopy (SEM). Secondly, cells potential to differentiate towards cardiac lineage was confirmed by the evaluation of cell surface markers (CD106, CD172A, CD36). RT-qPCR was performed to measure the increased expression of early cardiac mesoderm lineage genes (*ISL1, MESP1, KDR*), myogenic (*CNN1, DES*) and cardiomyogenic (*MEF2C, NKX-2.5, ACTN1, cTNT, cTNI*) differentiation genes, also genes that encode cardiac ion channels (*KCND3, KCNJ12, SCN5A, HCN2, CACNA1D*). Seeking to understand processes happening through the differentiation, various signaling and regulation protein-coding genes were also evaluated (*FOXO1, PDGFB, TGFBR1, mTOR, VEGFA, WNT4, Notch1*). Also, the impact of epigenetic modification, which is responsible for active chromatin state in cardiac-like cells (H3K9Ac) was investigated by chromatin immunoprecipitation assay, followed by RT-qPCR for promoter regions of *FOXO1, WNT4* and *RARβ* and *EZH2, EED* genes. To indicate changes at protein levels we performed the Western blot analysis for early cardiac transcription factor Nkx-2.5, forkhead protein FOXO3a, enhancer of zeste homolog 2 (EZH2), and modified histone H3K9Ac. Increased expression of Nkx-2.5 was additionally proved by immunofluorescence microscopy. Our research explores an action mechanism caused by Decitabine, Angiotensin II and TGF-β1 during the initiation of cardiomyogenic differentiation, and shows that differentiation process not only depends on the agent that was used, but also on the time of induction and most importantly, on the signaling pathway that certain cell type prefers to employ.

## Results

In current study, we explored the effects of 10 µM Decitabine, 1 µM Angiotensin II and 5 ng/mL TGF-*β*1 on initiation of cardiomyogenic differentiation of MenSCs, EndSCs, EmsSCs by analyzing cell morphology, surface markers, gene and protein expression levels and some gene expression associated with modified H3 at different points of treatment.

### Effects of decitabine, angiotensin II and TGF-β1 on MenSCs, EndSCs, EmsSCs morphology and cell surface marker expression

**Figure 1:**
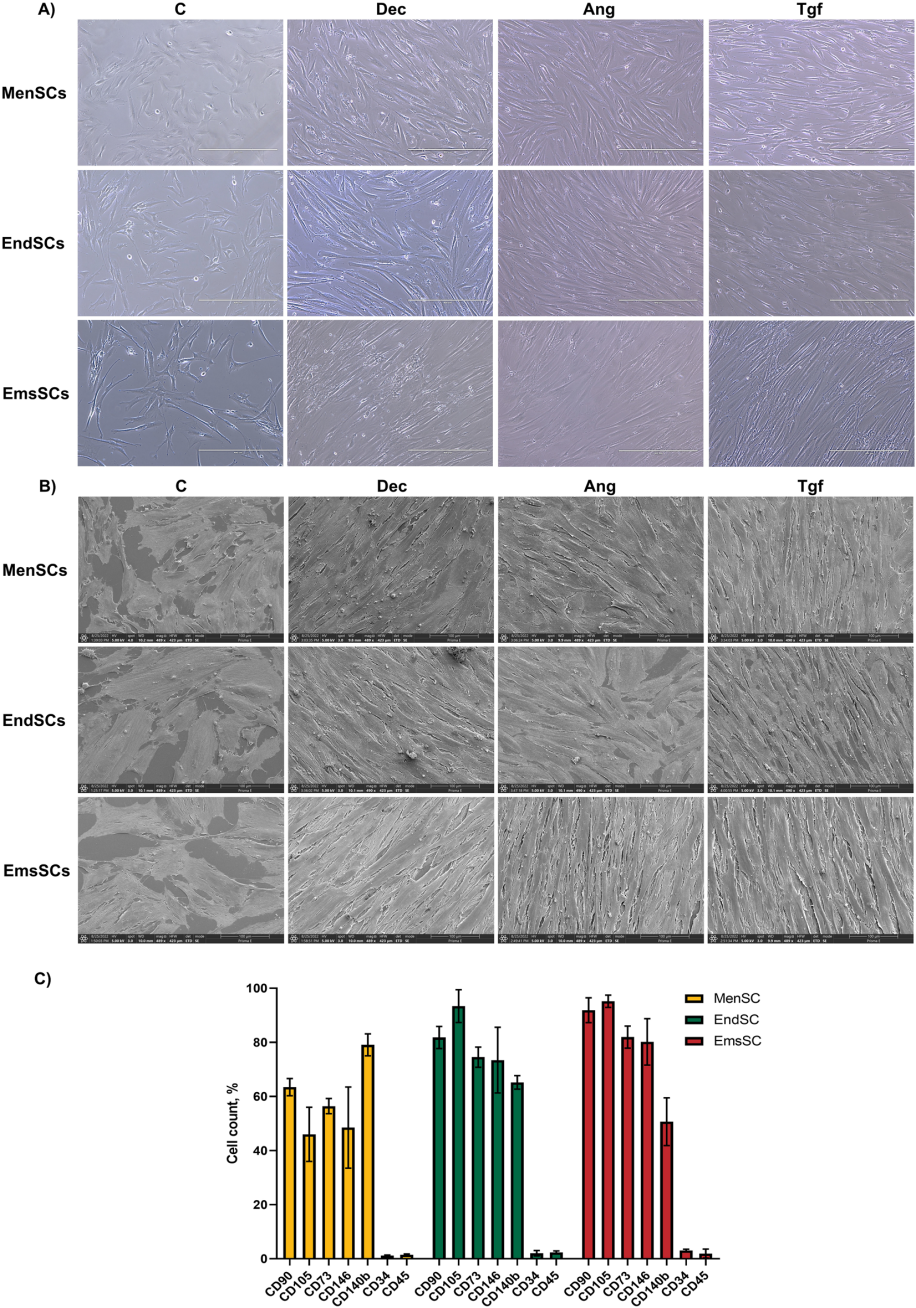
Morphology and CD markers of MenSCs, EndSCs and EmsSCs after 13 days of cardiomyogenic differentiation induction. C—control (untreated) cells, Dec – 10 µM Decitabine, Ang – 1 µM Angiotensin II, Tgf — 5 ng/mL TGF-*β*1. (**A**) Light microscopy images, scale bar 400 µm (10 × objective). (**B**) Scanning electron microscopy images, scale bar 100 µm (mag 489 ×). (**C**) Cell surface markers of endometrial origin SC.

To begin with, we examined the effects of cardiomyogenic initiators Decitabine, Angiotensin II and TGF-β1 on morphology and cell surface markers of MenSCs, EndSCs and EmsSCs after treatment. After 13 days of induction, morphological changes were observed in all studied SCs (Fig. 1). Induced cells were shown to be vastly elongated and branched out (Fig. 1B), with resemblance to cardiomyocyte-like cells.

**Figure 2:**
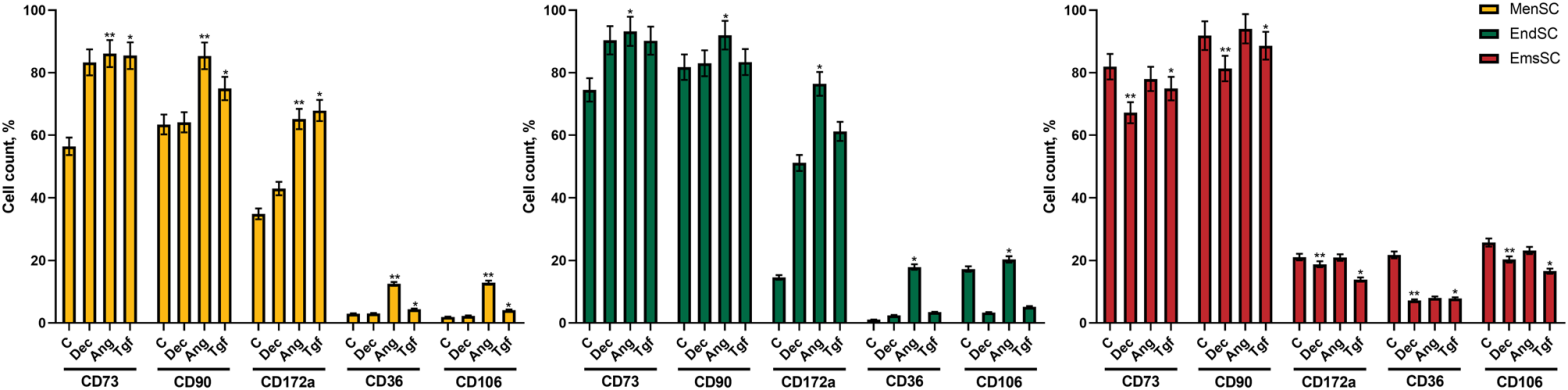
Cell surface markers of MenSCs, EndSCs and EmsSCs after 6 days of cardiomyogenic differentiation induction. The expression of mesenchymal cell surface markers CD73 and CD90; cardiomyogenic differentiation cell markers CD172a, CD36 and CD106 were measured using flow cytometry. C—control (untreated) cells, Dec – 10 µM Decitabine, Ang – 1 µM Angiotensin II, Tgf — 5 ng/mL TGF-*β*1. Results are presented as mean ± S.D. * represents a significant difference between control and treated cells with *p* < 0.05, ** represents a significant difference with *p* < 0.01, as calculated using ANOVA multiple comparison test.

Cell surface marker analysis revealed that MenSCs, EndSCs and EmsSCs are positive for mesenchymal cell markers CD73 and CD90. After 6 days of exposure to 1 µM Angiotensin II percentage of CD172a^+^, CD36^+^ and CD106^+^ MenSCs and EndSCs increased in comparison to control cells (Fig. 2). Decitabine and TGF-* β*1 increased percentage of CD172a^+^ MenSCs and EndSCs. However, cardiomyogenic differentiation initiators overall decreased the expression of CD172a, CD36 and CD106 in EmsSCs. As the expression of cardiomyocyte markers seem to differ between investigated cells, this suggests that MenSCs and EndSCs are more likely to change their cell surface marker expression into more cardiomyocyte-like state than EmsSCs, at least under these differentiation conditions.

### Specific gene expression changes are characteristic for induced cardiomyogenic differentiation of endometrium-derived stromal cells

**Figure 3:**
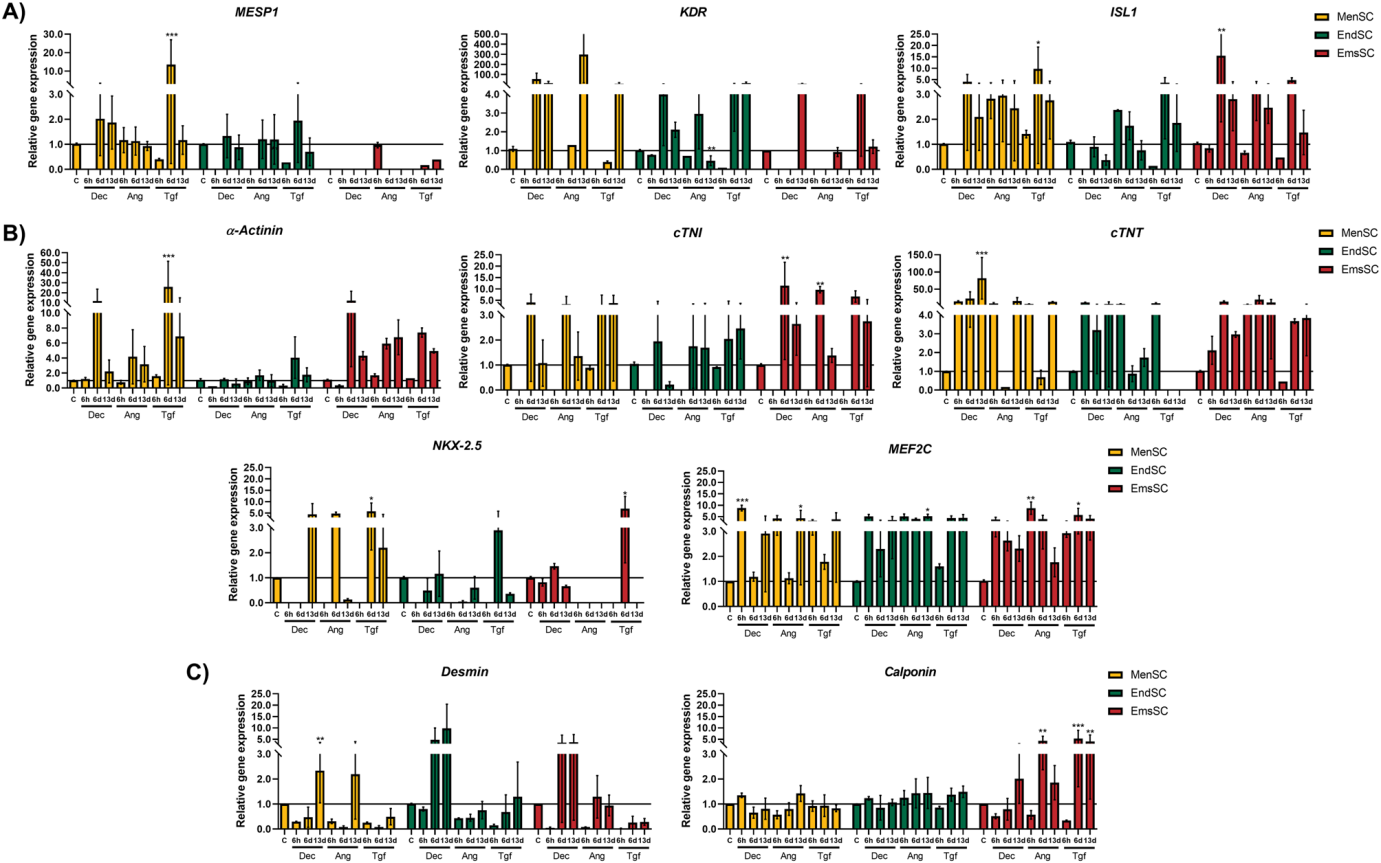
Real-time PCR analysis of expression of (**A**) cardiomyocite mesoderm genes, (**B**) cardiomyogenic differentiation genes, (**C**) myogenic differentiation genes. MenSCs, EndSCs, EmsSCs were treated with cardiomyogenic differentiation initiators for 6 h, 6 days and 13 days. C—control (untreated) cells, Dec – 10 µM Decitabine, Ang – 1 µM Angiotensin II, Tgf — 5 ng/mL TGF-*β*1. Results are presented as mean ± S.D. * represents a significant difference between control and treated cells with *p* < 0.05, ** represents a significant difference with *p* < 0.01, *** represents a significant difference with *p* < 0.001 as calculated using ANOVA multiple comparison test.

Further, we studied the expression of cardiomyogenesis related and signaling pathways gene expression after 6 h, 6 days and 13 days of MenSCs, EndSCs and EmsSCs exposure to cardiomyogenic differentiation initiators 10 µM Decitabine, 1 µM Angiotensin II and 5 ng/mL TGF-β1. Real-time PCR analysis of the expression of cardiomyocyte mesoderm genes (early differentiation genes) *MESP1, KDR, ISL1* showed that in MenSCs Decitabine and TGF-β1 overall increased expression of *MESP1, KDR* and *ISL1* after 6 days and 13 days of exposure, Angiotensin increased levels of *KDR* after 13 days of differentiation and *ISL1* expression increased after 6 h, 6 days and 13 days of MenSCs exposure to Angiotensin II. In EndSCs the most prominent *MESP1, KDR* and *ISL1* genes expression alterations were noticed after 6 days of induction with TGF-β1 – expression increased. Decitabine also increased the expression of *KDR* gene and Angiotensin II increased the expression of *KDR* and *ISL1* genes, but not *MESP1* gene after 6 days of treatment in EndSCs. In EmsSCs expression of *MESP1* remained stable throughout the differentiation with all agents, Decitabine and TGF-β1 increased the expression of *KDR* after 6 or 13 days of treatment and *ISL1* was upregulated after exposure to all 3 initiators after 6 and 13 days of differentiation (Fig. 3A).

Expression of *a-Actinin*, *cTNI* and *NKX-2.5* was shown to be upregulated in MenSCs after 6 days or 13 days of initiation with Decitabine, Angiotensin or TGF-β1 in MenSCs. *a-Actinin* expression remained stable in differentiation of EndSCs, whilst *cTNI* was upregulated after 6 days of Decitabine, Angiotensin and TGF-β1. *NKX-2.5* expression increased after 6 days of differentiation initiation with TGF-β1 in EndSCs and EmsSCs. *cTNT* and *MEF2C* genes were mostly upregulated in all studied cell cultures during cardiomyogenic differentiation after 6 h, 6 days and 13 days (Fig. 3B). Expression of *Desmin* was upregulated in MenSCs, EndSCs and EmsSCs after 6 or 13 days of cardiomyogenic differentiation induction with Decitabine, whilst other initiators were proven to be less effective. Expression of *Calponin* was not affected by differentiation initiators in MenSCs and EndSCs, whilst *Calponin* was upregulated in EmsSCs after 6 and 13 days of differentiation with Angiotensin and TGF-β1 (Fig. 3C).

**Figure 4:**
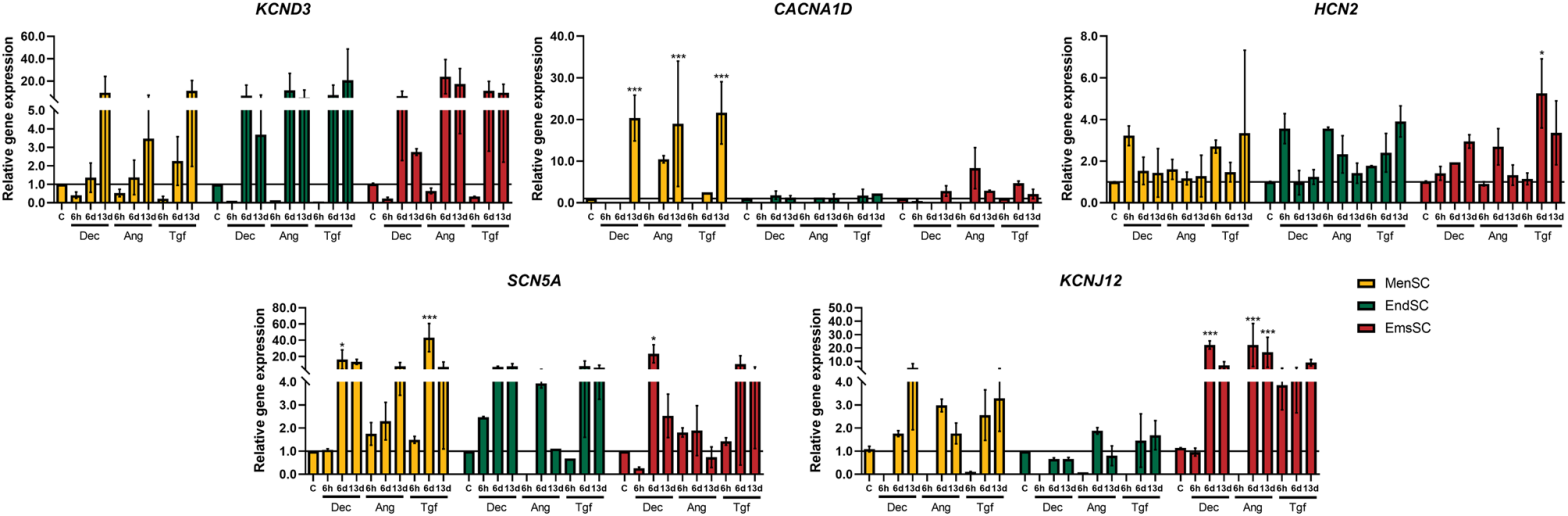
Real-time PCR analysis of expression of cardiac ion channels. MenSCs, EndSCs, EmsSCs were treated with cardiomyogenic differentiation initiators for 6 h, 6 days and 13 days. C—control (untreated) cells, Dec – 10 µM Decitabine, Ang – 1 µM Angiotensin II, Tgf — 5 ng/mL TGF-*β*1. Results are presented as mean ± S.D. * represents a significant difference between control and treated cells with *p* < 0.05, *** represents a significant difference with *p* < 0.001 as calculated using ANOVA multiple comparison test.

Additionally, we analyzed the expression of cardiac ion channels genes in MenSCs, EndSCs and EmsSCs during cardiomyogenic differentiation (Fig. 4). Expression of potassium voltage-gated channels genes *KCND3* and *KCNJ12* increased in MenSCs and EmsSCs after 6 and 13 days of initiation with Decitabine, Angiotensin and TGF-*β*1. Angiotensin and TGF-*β*1 had similar effect on potassium channels genes in EndSCs, whilst Decitabine did not increase *KCNJ12* expression in EndSCs. Expression on *CACNA1D* calcium voltage-gated channel gene was significantly upregulated in differentiated MenSCs and upregulated in EmsSCs, whilst remaining stable in EndSCs. Hyperpolarization activated cyclic nucleotide gated potassium and sodium channel 2 (*HCN2*) and sodium voltage-gated channel (*SCN5A*) genes were shown to be upregulated in induced to cardiomyogenic differentiation MenSCs, EndSCs and EmsSCs with all tested agents. Overall, data showed tendency that 6 and 13 days of treatment with Decitabine, Angiotensin II and TGF-*β*1 is more efficient period for upregulating the cardiac-related genes mentioned above, while 6 h of treatment seems to not be enough for cells to change its gene expression profile in most cases.

**Figure 5:**
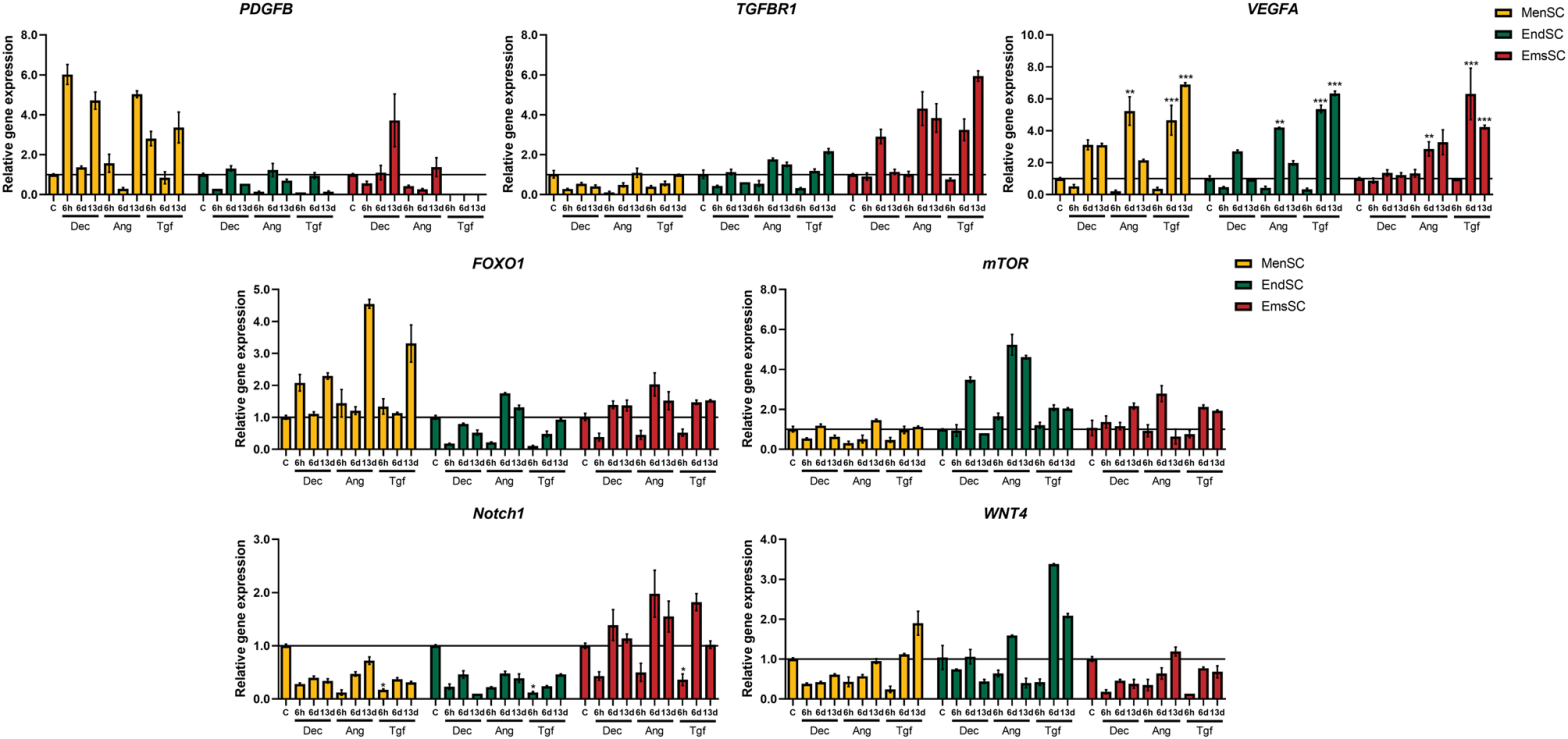
Real-time PCR analysis of expression of signalling pathways genes. MenSCs, EndSCs, EmsSCs were treated with cardiomyogenic differentiation initiators for 6 h, 6 days and 13 days. C—control (untreated) cells, Dec – 10 µM Decitabine, Ang – 1 µM Angiotensin II, Tgf — 5 ng/mL TGF-*β*1. Results are presented as mean ± S.D. * represents a significant difference between control and treated cells with *p* < 0.05, *** represent a significant difference with *p* < 0.001 as calculated using ANOVA multiple comparison test.

Due to varying signaling pathways possibly involved in induction of cardiomyogenic differentiation using Decitabine, Angiotensin II and TGF-β1 we investigated the expression of *PDGFB, TGFBR1, VEGFA, FOXO1*, *mTOR, Notch1* and *WNT4* expression levels (Fig. 5). We demonstrated that MAPK/ERK pathway gene *VEGFA* is upregulated in all endometrial derived stromal cells during initiation of cardiomyogenic differentiation, however, expression of *PDGFB* only increased in MenSCs and EmsSCs treated with Decitabine, whilst *TGFB1* expression was most prominent in induced EmsSCs. PI3/Akt pathway genes *FOXO1* and *mTOR* expression also varied between cell cultures – expression of *FOXO1* was increased in induced MenSCs, as it remained at around control levels in EndSCs and EmsSCs. We observed the highest *mTOR* expression level in EndSCs, slight increase in EmsSCs, but not in MenSCs. WNT signaling pathway gene *Notch1* was downregulated in MenSCs and EndSCs treated with Decitabine, Angiotensin II and TGF-β1, but upregulated in EmsSCs induced with all three agents. *WNT4* expression levels were affected only by TGF-β1 induction – expression of this gene increased in treated MenSCs and EndSCs after 6 or 13 days, compared to control. Such results suggest that investigated cells are prone to implement various downstream signals differently during differentiation – MAPK/ERK pathway related genes are mostly upregulated in MenSCs, PI3/Akt related in EndSCs, and both EndCSs and EmsCSs indicate the highest probability that the WNT signaling pathway is characteristic for induced cardiomyogenic lineage differentiation.

### Cardiomyogenic differentiation of endometrium-derived stromal cells effects on protein level changes

**Figure 6:**
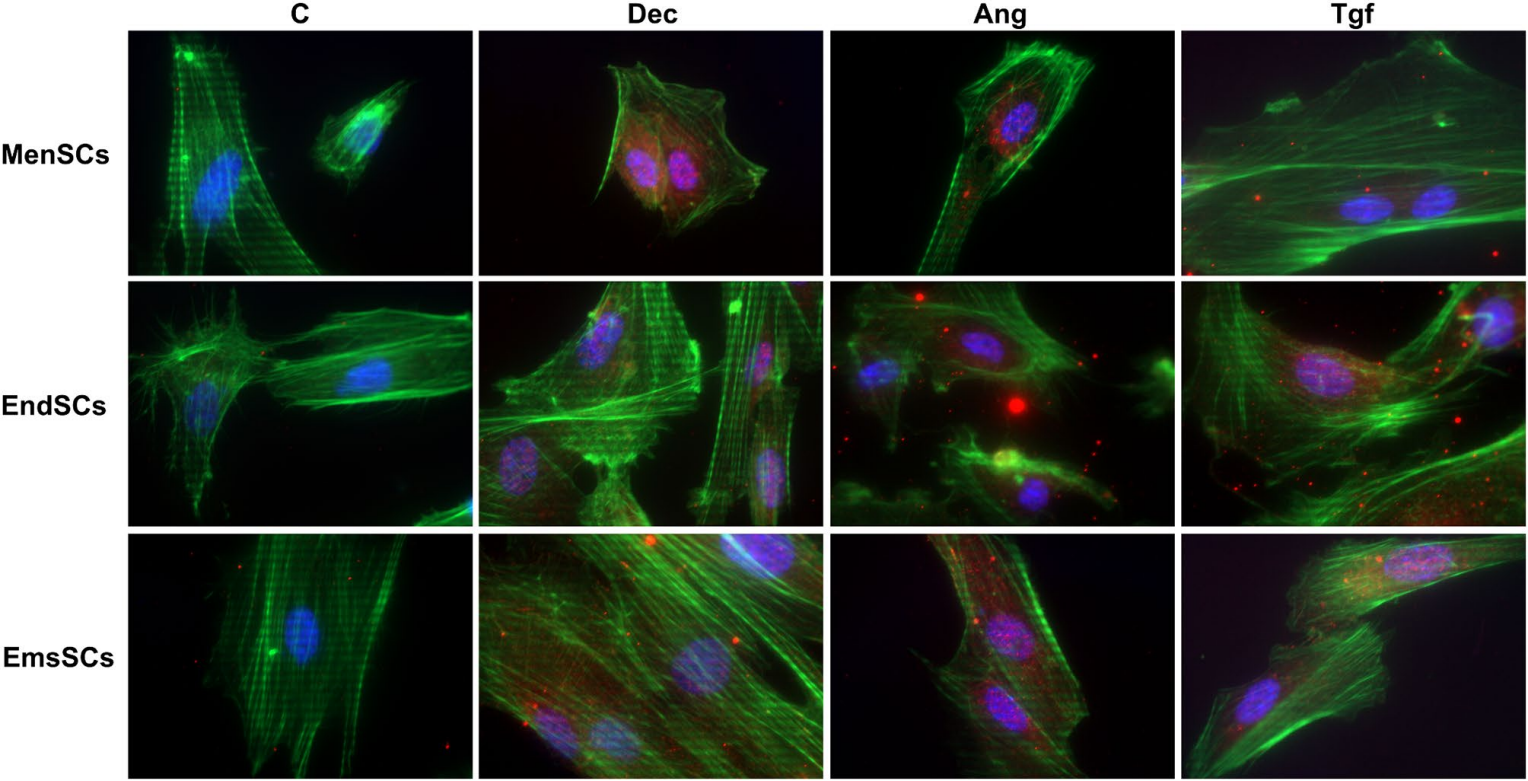
The levels and localization of Nkx-2.5 protein. MenSCs, EndSCs, EmsSCs were treated with cardiomyogenic differentiation initiators for 13 days. C—control (untreated) cells, Dec – 10 µM Decitabine, Ang – 1 µM Angiotensin II, Tgf — 5 ng/mL TGF-*β*1. Nuclei are blue, actin-phalloidin is green, and Nkx-2.5 is red. Samples were analysed using a Zeiss Axio Observer fluorescence microscope, with a 63 × objective in immersion oil. Scale bar = 10 µm.

In the study we examined protein expression changes of Nkx-2.5, early cardiac transcription factor participating in heart development, proteins involved in epigenetic regulation and signaling FOXO3a, EZH2 and histone H3K9Ac. Fluorescent imaging of Nkx-2.5 (Fig. 6) after 13 days of cardiomyogenic differentiation initiation with Decitabine, Angiotensin and TGF-* β*1 showed that levels of Nkx-2.5 increased in treated cells, however, effects seem to differ – in MenSCs fluorescence of Nkx-2.5 notably increased in Decitabine and Angiotensin II affected cells, but less so in TGF-β1 treated cells. In EndSCs fluorescence of Nkx-2.5 notably increased in Angiotensin II and TGF-β1 affected cells, but less so in Decitabine treated cells and in EmsSCs fluorescence of Nkx-2.5 increased compared to control, with similar effects of Decitabine, Angiotensin II and TGF-β1.

**Figure 7:**
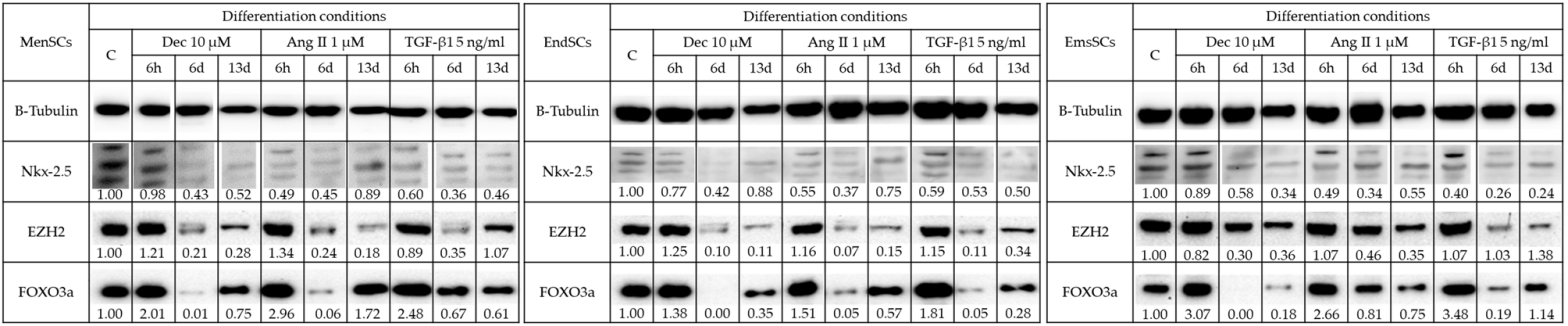
Western blot analysis of levels of cardiomyogenesis related proteins. MenSCs, EndSCs, EmsSCs were treated with cardiomyogenic differentiation initiators for 6 h, 6 days and 13 days. C—control (untreated) cells, Dec – 10 µM Decitabine, Ang – 1 µM Angiotensin II, Tgf — 5 ng/mL TGF-*β*1. B-Tubulin is control protein. Protein band intensity was calculated by the ImageJ program. Levels of each protein were determined according to the B-Tubulin. Samples derive from the same experiment and blots were processed in parallel. Displayed images are cropped, original full-length blots are presented in Supplementary Fig. 1A, B, C.

**Figure 8:**
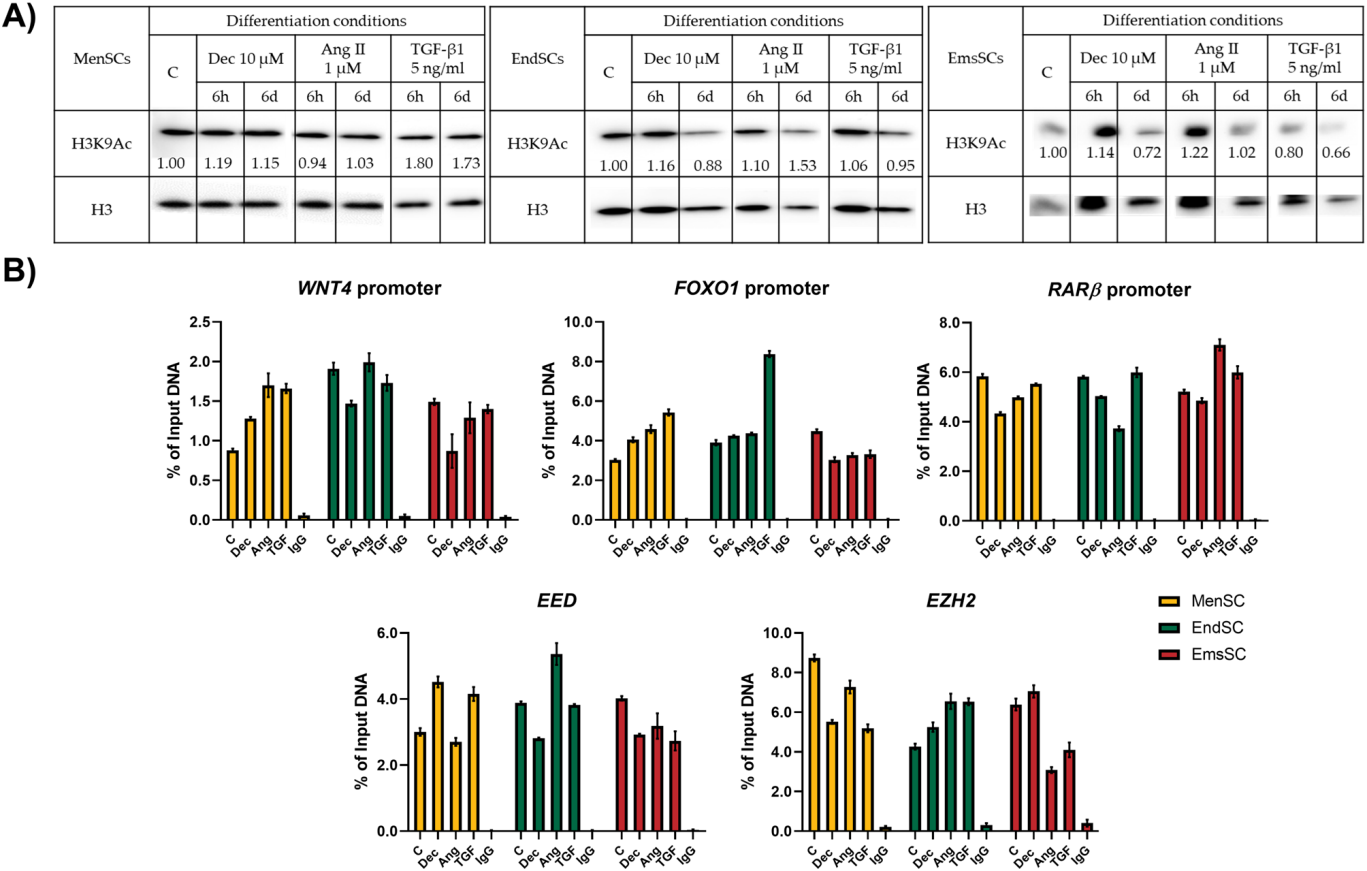
(**A**) Western blot analysis of levels of H3K9Ac. MenSCs, EndSCs, EmsSCs were treated with cardiomyogenic differentiation initiators for 6 h and 6 days. C—control (untreated) cells, Dec – 10 µM Decitabine, Ang – 1 µM Angiotensin II, Tgf — 5 ng/mL TGF-*β*1. H3 control protein. Protein band intensity was calculated by the ImageJ program, and the levels of protein calculated according to the H3. Samples derive from the same experiment and blots were processed in parallel. Displayed images are cropped, original full-length blots are presented in Supplementary Fig. 2. (**B**) ChIP-qPCR results of H3K9Ac changes in cardiomyogenesis related genes after 6 h of induced cardiomyogenic differentiation in MenSCs, EndSCs and EmsSCs. C—control (untreated) cells, Dec – 10 µM Decitabine, Ang – 1 µM Angiotensin II, Tgf — 5 ng/mL TGF-*β*1. Results are presented as mean ± S.D. Significance difference between control and treated cells evaluated using ANOVA multiple comparison test.

Western blot analysis (Fig. 7) revealed that Nkx-2.5 expression did not exceed control levels in MenSCs, EndSCs and EmsSCs. Same tendencies were observed in levels of EZH2 – after 6 h of differentiation induction with Decitabine, Angiotensin II and TGF-*β*1 were highest, without notably surpassing control cell levels. Levels of FOXO3awere also highest after 6 h of differentiation initiation. In EmsSCs levels of FOXO3a increased 3 times, in MenSCs around 2 times and in EndSCs 1,5 times compared to control. H3K9Ac levels (Fig. 8A) remained stable in MenSCs, EndSCs and EmsSCs during 6 h and 6 days of differentiation initiation with Decitabine and Angiotensin II, whilst exposure to TGF-* β*1 had varying effects: in MenSCs H3K9Ac levels increased, in EndSCs H3K9Ac levels remained unchanged and in EmsSCs H3K9Ac levels decreased after 6 h and 6 days compared to control. Taken together, changes in cardiac differentiation related protein levels tend to be more notable in earlier differentiation stages (6 h, 6d), than after 13 days of initiation.

### H3K9Ac modification changes in cardiomyogenic diferentiation-associated gene regions of MenSCs, EndSCs and EmsSCs

We demonstrated different levels of H3K9 acetylation during TGF-β1 initiated endometrial origin SCs cardiomyogenic lineage differentiation, therefore, cardiomyogenesis related genes regions were examined for H3K9Ac changes performing ChIP-qPCR after 6 h of initiation (Fig. 8). Although no significant changes were detected, the tendency for H3K9Ac increased association was observed in MenSCs *WNT4* promoter, MenSCs and EndSCs *FOXO1* promoter and EndSCs *EZH2* gene. H3K9Ac levels in *WNT4* and *FOXO1* promoter decreased in treated EmsSCs. Levels of H3K9Ac in RARβ promoter region varied – in cardiomyogenic differentiation induced MenSCs and EndSCs levels fluctuated around control or decreased whilst in Angiotensin II and TGF-β1 treated EmsSCs H3K9Ac levels increased compared to control. In *EED* gene region levels of H3K9Ac increased after Decitabine, TGF-β1 and Angiotensin treatment of EndSCs, however, in EmsSCs the association of H3K9Ac in *EED* gene region was decreased. As the changes are only mild in most cases, it is quite important to notice that differentiating cells keep expressing those genes without major diminutions as H3K9Ac is attributed to be one of the epigenetic modifications that is necessary for keeping active chromatin state in cardiac-like cells.

## Discussion

Cardiovascular disease is one of the main reasons for death in Europe with around 1.8 mln. Deaths per year in the EU^11^. Due to its high morbidity and mortality rates, loads of efforts and resources are directed towards researching cardiovascular diseases, its mechanisms, signaling pathways and treatment strategies. Therefore, understanding of cardiovascular development is key to creating modern therapy options. Detailed protocols are created to induce cardiomyogenic differentiation of human induced pluripotent stem cells (hiPSC) to ventricular cardiomyocytes (markers *TNNT2, MYL2, IRX4*), atrial cardiomyocytes (markers *TNTT2, NNPA, KCNJ3, NR2F2, SLN, CACNA1D, TBX5, MYL7, KCNA5*) and sinoatrial cardiomyocytes (markers *NKX-2.5, SHOX2, HCN4, TBX3, ISL1, KCNJ3, TBX18, COUPTFII*)^12^. However, due to deficient differentiation and maturation of induced cardiomyocytes and debate over teratogenic potential and safety of induced pluripotent stem cells^13,14^, cardiomyogenic differentiation mechanisms and potential of adult stem cells, for example mesenchymal stromal cells should be considered.

In this study, we explored the effect of the three inducers – Decitabine, Angiotensin II and TGF-*β*1, on the expression of various molecular factors during induced early (6 h, 6 days and 13 days) cardiomyogenic differentiation of menstrual stromal cells, endometrium stromal cells and endometriosis stromal cells. In this research we use term “stromal cells” as the cell populations used by us in the study did not demonstrate high specificity (over 95%) cell surface markers’—CD73, CD90, CD105, characteristic for stem cells. Stromal cells were characterized by the expression of CD90, CD106, CD73, CD146 and CD140b, though due to usage of primary cultures and stromal cells of early passages the expression of most markers did not exceed 95%. Lundy and colleagues showed that BMP-4 induced embryonic stem cells after 14 days in culture start to be contractile, and after 20 days, cells become larger, elongated, and percentage of cardiomyocytes in culture reaches 86%^15^. Human induced pluripotent stem cells can develop into beating cardiomyocytes after 10 days in culture, thus it requires utilization of wide range of inhibitors and it takes about 30 days to reach fully mature cardiomyocytes^16,17^.

We observed that after 13 days of induction with Decitabine, Angiotensin II and TGF-β1, endometrial origin stromal cells elongate, branch out and connect. This we confirmed by two different methods – by using light microscopy and scanning electron microscopy. We also demonstrated that after 6 days of exposure to 1 µM Angiotensin II percentage of CD172a^+^, CD36^+^ and CD106^+^ MenSCs and EndSCs increased compared to control. CD172a is expressed in human heart, therefore, iPSCs-derived cardiomyocytes are characterized by CD172a, CD106 (VCAM1) and *cTNT* expression and CD36 for maturation^18,19^. However, mesenchymal stromal cells are less potent in differentiation than hiPSC, therefore in 13 days of cardiac lineage differentiation initiation none of the positive CD markers reached the level of 95%^20^ and there was no spontaneous beating registered in any analyzed cell cultures, meaning that researched cell cultures did not differentiate into mature cardiomyocytes in 2 weeks.

During mesoderm specification to cardiac lineage, induction with BMP4, Activin A, WNT3A, inhibition of Notch signaling pathway activates *MESP1* expression (days 1–2 of differentiation), *KDR* expression (days 3–8 of differentiation), and triggers downstream cardiac precursors *GATA4*, *MEF2c, HAND2* and *NKX-2.5*^21,22^. Further down cardiac differentiation pathway late cardiomyocytes express *cTNT, a-Actin, MLC2a, MLC2v, SCN5A, CACNA* and *IRX4*^23^. In our study expression of *MESP1, KDR* and *ISL1* increased in induced MenSCs and EndSCs after 6 days and 13 days of exposure, however, in EmsSCs expression of *MESP1* remained stable throughout the initiation of differentiation with all initiators, but *KDR* and *ISL1* was upregulated after exposure to all 3 initiators after 6 and 13 days of differentiation. We also demonstrated tendency of upregulation of *a-Actinin, cTNI, cTNT*, *MEF2C* and *NKX-2.5* in induced endometrial-origin stromal cells, though expression levels varied between cell cultures. Role of cardiac cells transmembrane ion channels is critical for heart function, with abnormalities causing heart problems, such as arrhythmia, therefore emphasizing the importance of expression of cardiac ion channels genes^24^. In this study we showed increase of expression in *KCND3*, *KCNJ12, HCN2, SCNA5* genes in endometrium-derived stromal cells after 6 and 13 days of initiation with Decitabine, Angiotensin and TGF-*β*1, but not in *CACNA1D* gene in EndSCs. Expression of L-type calcium channels (*CACNA1C*, *CACNA1D*) is characteristic to left ventricular cardiac tissue^25^.

Proper regulation of various signaling pathways is proven to be essential for cardiomyocyte differentiation. In 2021 study of mouse embryonic stem cells Wang and colleagues showed importance of MAPK/ERK signaling pathway by showing that Igf2 or Igf1r deletion suppresses cardiomyocyte differentiation^26^. We demonstrated that MAPK/ERK pathway genes *VEGFA* and *PDGFB* are most prominently upregulated in early cardiomyogenic differentiation induced MenSCs. Importance of PI3/Akt signaling pathway was proven by studying importance of CCN2 to cardiomyocytes, it was shown that increased levels of CCN2 enhances tolerance to oxidative stress in cardiomyocytes through phosphorylation of Akt and GSK-3β in PI3/Akt/GSK-3β signaling^27^. Moreover, PI3/Akt signaling was proven to be critical for promotion of cardiomyocyte proliferation and survival via upregulation of Yes-associated protein dependent reporter gene expression^28^. Most of hiPSC differentiation to cardiomyocytes protocols are based on alterations of WNT pathway by using various inhibitors, with studies showing time of WNT pathway manipulation being crucial step in success of cardiac differentiation^14^. Lu and others have made the point that WNT signaling possibly promotes *ISL1* expression together with upregulated histone H3K9 acetylation, which coincide with our research as we have also detected increased *ISL1* gene expression during differentiation^29^. We demonstrated that during Decitabine, Angiotensin II and TGF-β1 induced cardiomyogenic lineage differentiation, PI3/Akt related genes (*mTOR*) are mainly upregulated in EndSCs, and WNT pathway genes (*Notch1, WNT4*) in EndCS and EmsCS.

Epigenetic regulation is known to play role in cardiac differentiation, histone acetyl transferase Gcn5, histone deacetylase SIRT6, histone methyltransferase PRC2 and histone demethylase UTX affect cardiac genes expression and regulate differentiation^30^. Studies of inhibition of HAT in cardiomyocytes showed significant decrease in acetylation of H3 in promoter regions of cardiomyogenesis related genes^31^. Inhibition of EZH2 is shown to activate cardiac genes in somatic cells induced to cardiomyocytes^32^. Our study as well revealed the decrease of EZH2 protein after 6 and 13 days of differentiation initiation, especially after exposure to Decitabine and Angiotensin II. Moreover, EZH2 is shown to downregulate expression of OCT-4 during embryonic cell cardiomyogenic differentiation^33^. Many different epigenetic modifications related cardiomyogenesis have been studied, including repressed chromatin (H3K27me3, H3K9me3) and active-state chromatin (H3K4Me3, H4HyperAc, H3K9Ac) marks^34^. We demonstrated different levels of H3K9Ac change during TGF-β1 initiated MenSCs, EndSCs and EmsSCs early cardiomyogenic lineage differentiation, in MenSCs *WNT4* promoter, MenSCs and EndSCs *FOXO1* promoter and EndSCs *EZH2* gene regions the level of H3K9Ac increased. H3K9Ac levels in *WNT4* and *FOXO1* promoter regions decreased in treated EmsSCs.

**Figure 9:**
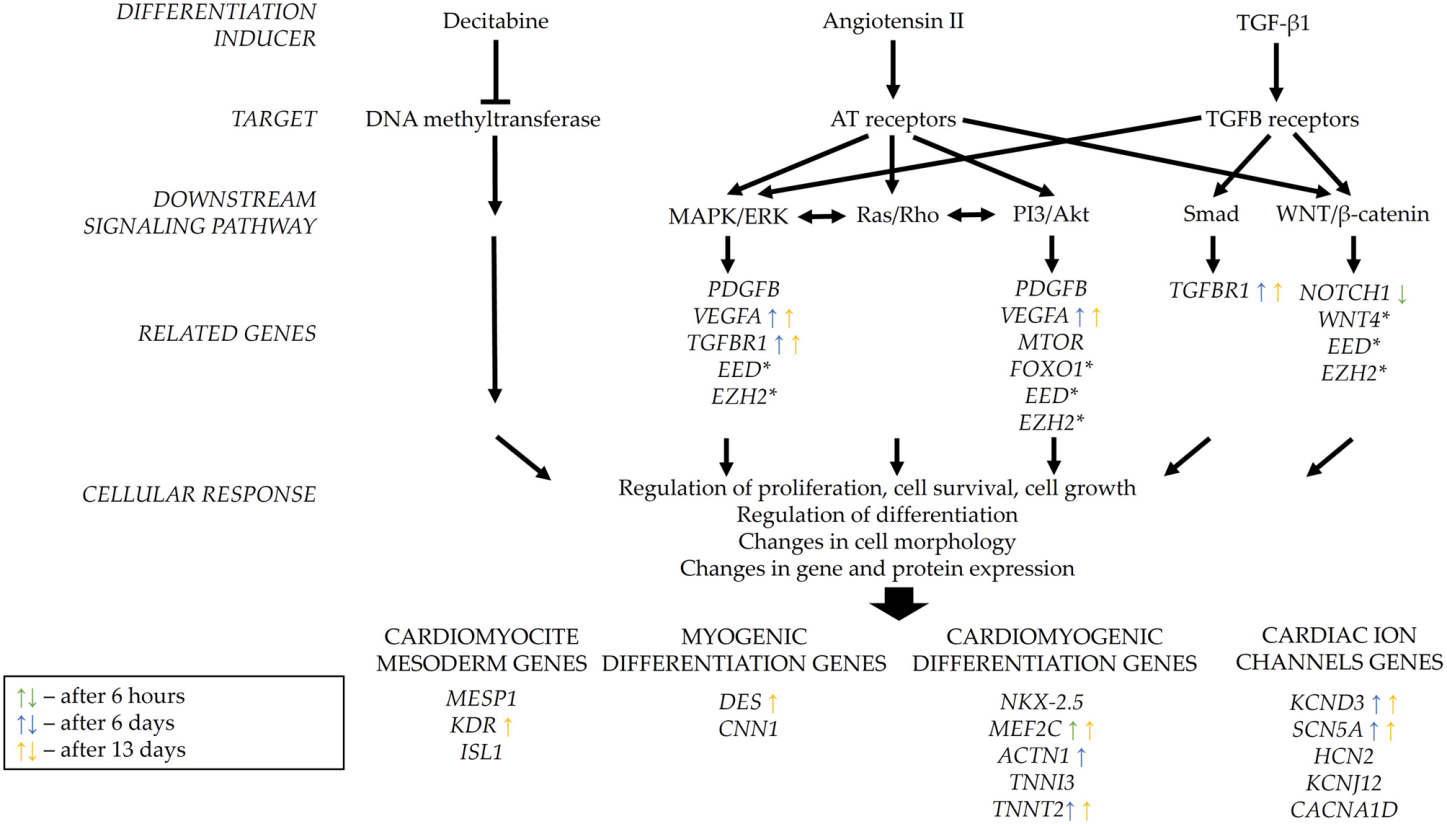
Schematic overview of the molecules investigated in this study, that are involved in main signalling pathways related to MenSCs, EndSCs and EmsSCs cardiomyogenic differentiation. * denotes genes that were measured after chromatin immunoprecipitation assay targeted to H3K9Ac modification. Arrows show main tendencies of gene expression changes after 6 h (green), 6 days (blue), or 13 days (yellow) of cardiomyogenic differentiation.

Overall, our study (Fig. 9) shows that Decitabine, Angiotensin II and TGF-β1 can induce early stages of cardiomyogenic lineage differentiation in endometrium-derived stromal cells (MenSCs, EndSCs and EmsSCs) but separate cell cultures seem to response to treatment a bit differently. Analysis of signaling pathways genes showed that mainly MAPK/ERK, PI3/Akt, and WNT signaling pathways are activated during the differentiation initiation. That implies that Angiotensin II would be the most potent initiator, as it not only induces MAPK/ERK and PI3/Akt signals itself but can also affect the production of TGF-β molecules in differentiating cell, this way adding the possibility of successful differentiation in TGF-β mediated signaling pathways as well^8^. The role of Angiotensin II was also shown in embryonic stem cells, where it promoted cardiac differentiation via ERK, p38 and JNK pathways, as it has been assessed by the detection of activated kinases. As these pathways are activated by signals conveyed through angiotensin type 1 receptor (ATR1) mainly, its agonist Angiotensin II should play a key role in the differentiation process^35^. Newest studies also confirm the connection between ATR1 and TGF-β receptors in cardiac myofibroblast differentiation, this way supporting the Angiotensin II ability to activate differentiation processes^36^.

Differention-induced MenSCs and EndSCs showed high levels of CD172a, they strongly expressed Nkx-2.5 together with most noticeable changes in other-cardiac like molecules, while EmsCS seemed to be less affected by differentiation agents in some cases. It could be due to the pathological source of the EmsSCs (endometriosis tissue) and their mediated signaling mechanisms, but more studies should be conducted for further investigation. Other limitations of this study include lack of functional assays, such as determination of cardiac specific ion channels functionality, action potential, calcium, contraction assessment of induced cells and shortage of in vivo studies to examine cell faith, since it was previously shown that stromal cells can stimulate angiogenesis and endogenous myocardial regeneration^37^. On the other hand, MenSCs and EndSCs demonstrated a promising perspective to differentiate into mature cardiomyocytes as the proper treatment circumstances will be eventually determined, including exposure time, combinations of initiators together with functional features of differentiated cells.

## Materials and methods

### Human endometrium origin stem cells isolation and cultivation

Endometrium-derived stromal cells were isolated from a) endometrium scratch, b) endometriosis lesion and c) menstrual blood with approval of the Lithuanian Ethics Committee (No. 158200–18/7–1049-550) in accordance with the Declaration of Helsinki. Endometrium samples were collected by medical professionals as a part of assisted reproductive technologies (ART), endometrial lesion samples were obtained by medical professionals during planed endometriosis operations. Stromal cells from endometrium and endometriosis tissue were isolated and cultivated as described earlier^38^. Menstrual blood samples were collected using a DivaCup during the second or third day of menses. Menstrual stromal cells were isolated and cultivated as described earlier^39^.

### Induction of cardiomyogenic lineage differentiation

Cardiomyogenic lineage differentiation of endometrium-derived stromal cells was induced with 1 µM Angiotensin II (Sigma-Aldrich Chemie GmbH, Taufkirchen, Germany), 10 µM Decitabine (Sigma-Aldrich Chemie GmbH, Taufkirchen, Germany), and 5 ng/mL TGF-*β*1 (R&D Systems, MN, USA). Basal differentiation medium consisted of DMEM 1 g/L glucose (Gibco, Thermo Fisher Scientific, Waltham, MA, USA), 10% FBS (Gibco, Thermo Fisher Scientific, Waltham, MA, USA), 100 μg/ml streptomycin and 100 U/ml penicillin (Gibco, Thermo Fisher Scientific, Waltham, MA, USA). Angiotensin II and TGF-*β*1 was added every 3 days for whole differentiation period. Decitabine was added for 24 h and then basal differentiation medium enriched with 10% horse serum (Gibco, Thermo Fisher Scientific, Waltham, MA, USA) was added every 3 days for whole differentiation period. Differentiation was analyzed after 6 h, 6 days and 13 days. Control group were undifferentiated cells of each cell culture. Number of biological replicates – 3 for each group, technical replicates – 2, cells in passages 4 and 5.

### Scanning electron microscopy

Endometrium-derived stromal cells were seeded on sterilized chambered-coverslips in DMEM/F12 (Gibco, Thermo Fisher Scientific, Waltham, MA, USA) supplemented with 10% Fetal Bovine Serum (FBS) (Gibco, Thermo Fisher Scientific, Waltham, MA, USA), 100 U/mL penicillin and 100 µg/mL streptomycin (Gibco, Thermo Fisher Scientific, Waltham, MA, USA) and incubated overnight to adhere to the coverslip. After the incubation, the medium was aspired and 2 mL of 2.5% glutaraldehyde solution (Sigma-Aldrich, St. Louis, MO, USA) was added and incubated at room temperature for 60 min. Samples were washed with distilled water for 10 min three times. Then, 2 mL of 2% osmium tetroxide solution (Sigma-Aldrich, St. Louis, MO, USA) was added and incubated for 20 min at room temperature. Afterwards, samples were washed with distilled water for 10 min three times. Samples were dehydrated using ethanol gradient: incubation of 10 min using each concentration: 25%, 50%, 75% and 96% without drying completely. Samples were dried using a critical point dryer (K850, Quorum Technologies, Lewes, United Kingdom) and covered with a 10 nm thick silver layer using a sputter coater (Q150R, Quorum Technologies, Lewes, United Kingdom). The samples were examined by a scanning electron microscope (PrismaE, Thermo Fisher Scientific, Eindhoven, The Netherlands). Number of biological replicates – 2 for each group, technical replicates – 2, cells in passage 7.

### Human endometrium origin stromal cells’ surface markers analysis

Endometrium-derived stromal cells surface markers were evaluated by flow cytometry. Briefly, 0.05 × 10^6^ cells per assay were collected by centrifugation at 500 × *g* for 5 min. Cell pellet was washed twice in PBS with 1% bovine serum albumin (BSA) (Sigma-Aldrich, St. Louis, MO, USA). Cells were suspended in 50 μL of PBS containing 1% BSA and incubated with the antibodies against cell surface markers (Table 1) in the dark at 4 °C for 30 min. Antibody dilution 1:25. Antibodies are presented in Table S1. After incubation samples were washed with PBS with 1% BSA, cells suspended in 200 μL PBS with 1% BSA and analysed using Guava easyCyte 8HT flow cytometer (Millipore, Burlington, MA, USA) using GuavaSoft 3.3 software. Number of biological replicates – 3 for each group, technical replicates – 2, cells in passage 5.

**Table 1:** Antibodies used in cell surface marker analysis.

Antibody/Fluorophore	Isotype	Manufacturer
CD172a/FITC	IgG2a, κ	Biolegend, San Diego, CA, USA
CD106/APC	IgG1, κ	Biolegend, San Diego, CA, USA
CD36/APC	IgG2a, κ	Biolegend, San Diego, CA, USA
CD105/APC	IgG2a, κ	Exbio, Vestec, Czech Republic
CD146/APC	IgG1, κ	Biolegend, San Diego, CA, USA
CD140b/APC	IgG1	Exbio, Vestec, Czech Republic
CD34/FITC	IgG2a, κ	Biolegend, San Diego, CA, USA
CD45/FITC	IgG1, κ	BD Pharmingen, San Jose, CA, USA
CD90/APC	IgG1, κ	Exbio, Vestec, Czech Republic
CD73/FITC	IgG1, κ	Exbio, Vestec, Czech Republic

### Gene expression analysis by RT-qPCR

Total RNA from endometrium-derived stromal cells was purified using TRIzol reagent (Invitrogen, Carlsbad, CA, USA), cDNA was synthesized using LunaScript® RT SuperMix Kit (New England Biolabs, Ipswich, MA, USA), and qPCR was performed using Luna® Universal qPCR Master Mix (New England Biolabs, Ipswich, MA, USA) on the RotorGene 6000 system (Corbett Life Science, QIAGEN, Hilden, Germany). Primer sequences (Metabion international AG, Planegg/Steinkirchen, Germany) are presented in Supplementary Table S1. mRNA levels were normalized to *GAPDH* expression. Relative gene expression was calculated using the ΔΔCt method. Number of biological replicates – 3 for each group, technical replicates – 3, cells in passage 6.

### ChIP-qPCR

Endometrium-derived stromal cells’ ChIP assay was performed using Zymo-Spin ChIP Kit (Zymo Research, Irvine, CA, USA) according to modified manufacturers’ instructions. Stromal cells were cross-linked in the culture flasks by adding 1% final concentration formaldehyde into the medium and incubated for 10 min at 37 °C. 0.125 M final concentration of glycine solution was added to the flasks to terminate cross-linking. After fixation, cells were prepared using Zymo-Spin ChIP kit (Zymo Research, Irvine, CA, USA), and sonicated using a Bioruptur® Pico sonicator (Diagenode, Liege, Belgium) with an average fragment size of 200–600 bp. The sheared chromatin was immunoprecipitated with an IgG-control antibody (Advansta, San Jose, CA, USA) and antibody against Acetyl-Histone H3 Lys9 (Cell Signalling Technology, Danvers, MA, USA) for 24 h at 4 °C. Precipitated DNA was analysed by qPCR with genomic DNA specific primers (Supplementary Table S2.). Input sample represented a 10% fraction of the total chromatin used in each immunoprecipitation. The percentage of the enrichment of histone modification on a specific gene region was calculated using percent input method. Number of biological replicates – 3 for each group, technical replicates – 3, cells in passage 6.

### Fluorescent microscopy

Endometrium-derived stromal cells fixed on coverslips for 15 min with PBS + 4% paraformaldehyde solution at RT. Samples were washed with PBS and permeabilized using 10% Triton X-100/PBS for 20 min, then washed in PBS again. Cells were blocked with PBS + 2% BSA for 30 min at 37 °C. To detect expression of Nkx-2.5 the coverslips were incubated with primary antibodies against Nkx-2.5 and secondary goat anti-rabbit IgG, Alexa Fluor-594 antibody (Thermo Fisher Scientific, IL, USA) in a humid chamber for 1 h at 37 °C. Actin-phalloidin was labelled with Alexa Fluor-488 Phalloidin (Thermo Fisher Scientific, IL, USA) for 30 min at RT in a humid chamber. After each incubation coverslips were washed with PBS + 1% BSA. Nuclei were stained with 300 nM DAPI (Invitrogen, OR, USA). Coverslips were mounted using Dako Fluorescent Mounting Medium (Agilent Technologies, CA, USA). Samples were analysed using Zeiss Axio Observer (Oberkochen, Germany) fluorescent microscopy system with the 63 X objective with immersion oil and Zen BLUE 12.0 software. Number of biological replicates – 2 for each group, technical replicates – 2, cells in passage 9.

### Western blot analysis

Proteins from endometrium-derived stromal cells were fractionated on a 7–15% polyacrylamide gradient SDS/PAGE gel using Tris–glycine buffer. The PVDF membrane (Immobilon P; Millipore, Billerica, MA, USA) were probed with the primary antibodies β-Tubulin, Nkx-2.5, EZH2, FOXO3a, H3K9Ac, H3 according to manufacturer’s instructions. The membrane was subsequently washed four times with PBS–Tween-20 and then incubated with horseradish-peroxidase (HRP)-linked secondary corresponding antibody for 1 h at RT. Βeta-Tubulin and H3 were used as loading control. Chemiluminescent signal detection was carried out on ChemiDoc XRS + System (BIORAD, Hercules, CA, USA). Quantitative evaluation was performed using ImageJ software. Number of biological replicates – 2 for each group, technical replicates – 3, cells in passage 6.

### Statistical analysis

Statistical analysis was performed using GraphPad Prism version 8.0.1 for Windows, GraphPad Software (San Diego, CA, USA). Data in graphs are represented as mean ± standard deviation (S.D.). The statistical significance of difference of means of groups was calculated using the Anova multiple comparison test; significance was set at p ≤ 0.05 (*), p ≤ 0.01 (**), and p ≤ 0.001 (***).

### Ethical approval

The study was conducted in accordance with the Declaration of Helsinki and approved by the Lithuanian Ethics Committee (No. 158200–18/7–1049-550).

### Informed consent

Informed consent was obtained from all subjects involved in the study.

### Supplementary Information

Supplementary Information.

## Data Availability

The data presented in this study are available on request from the corresponding author.
