# Supplementary material for: Molecular changes in endometrium origin stromal cells during initiation of cardiomyogenic differentiation induced with Decitabine, Angiotensin II and TGF- β1
Source: Sci Rep. 2024 Jul 23;14:16966. doi: 10.1038/s41598-024-68108-0 (PMC11266582; doi:10.1038/s41598-024-68108-0)
Supplement: Supplementary file 1 — Supplementary Information. [file 41598_2024_68108_MOESM1_ESM.pdf]

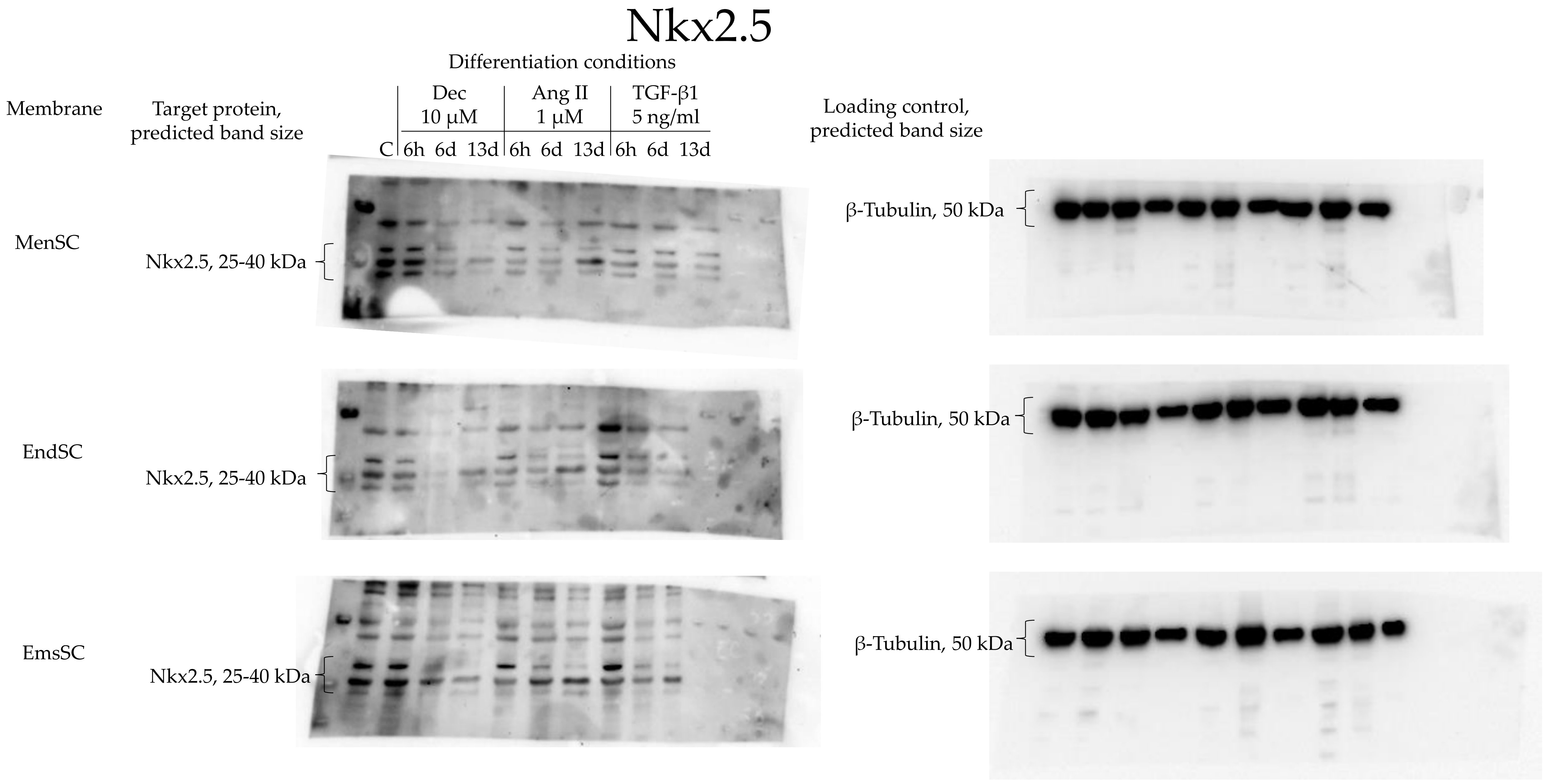

Supplementary figure 1A. Western blot analysis of levels of NKX2,5

EZH2

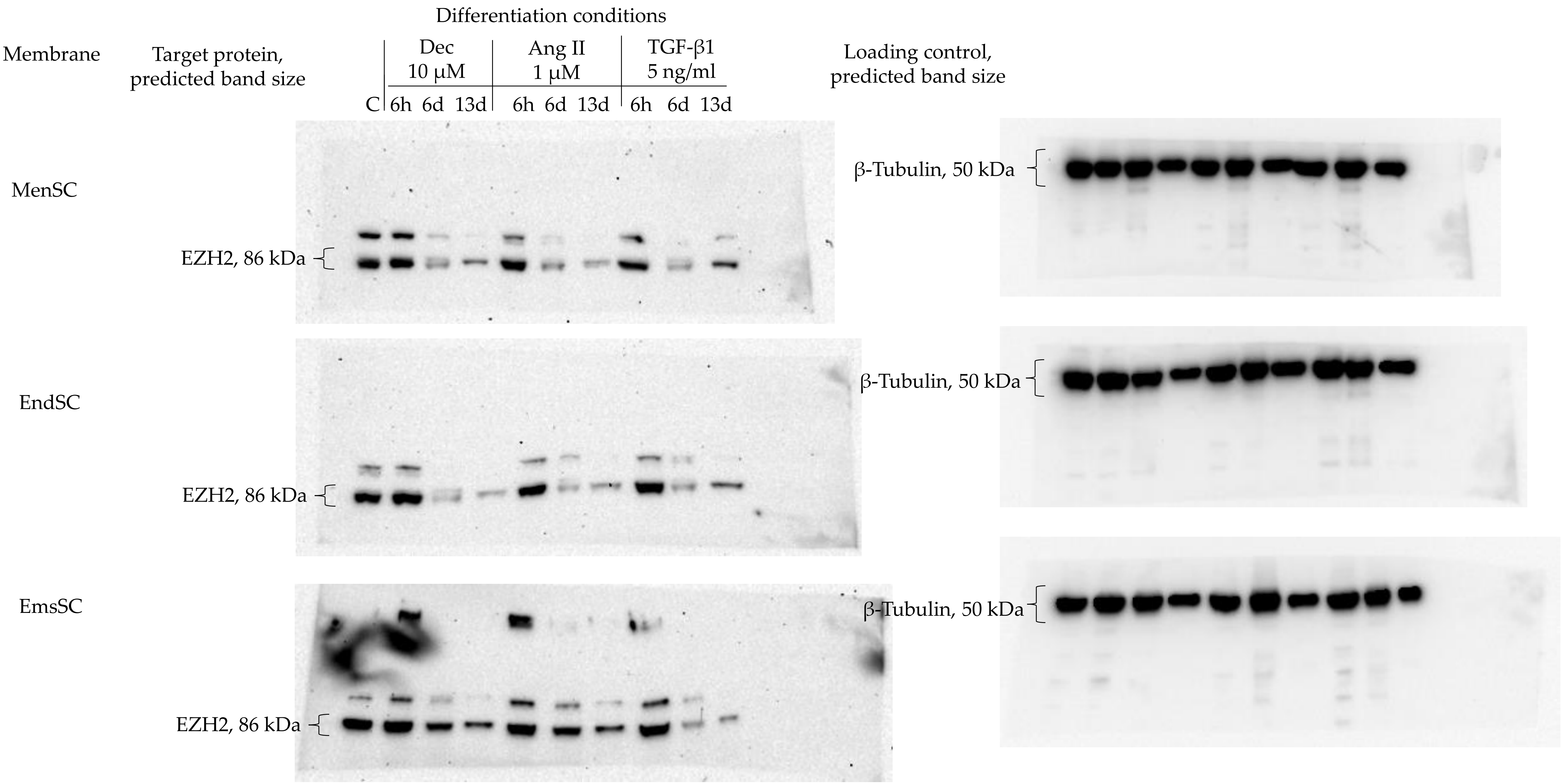

Supplementary figure 1B. Western blot analysis of levels of EZH2

FOXO3a

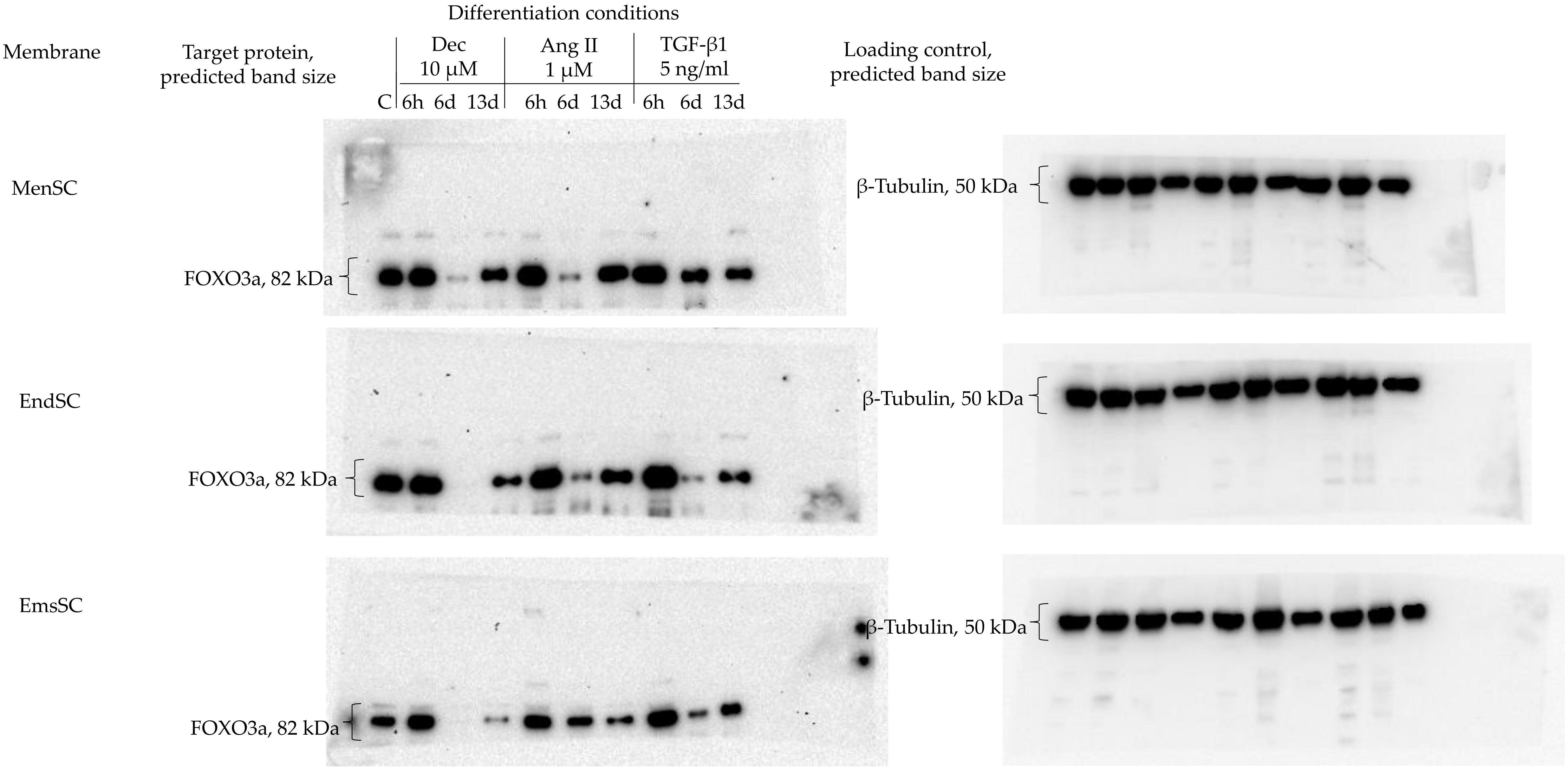

Supplementary figure 1C. Western blot analysis of levels of FOXO3a

H3K9Ac

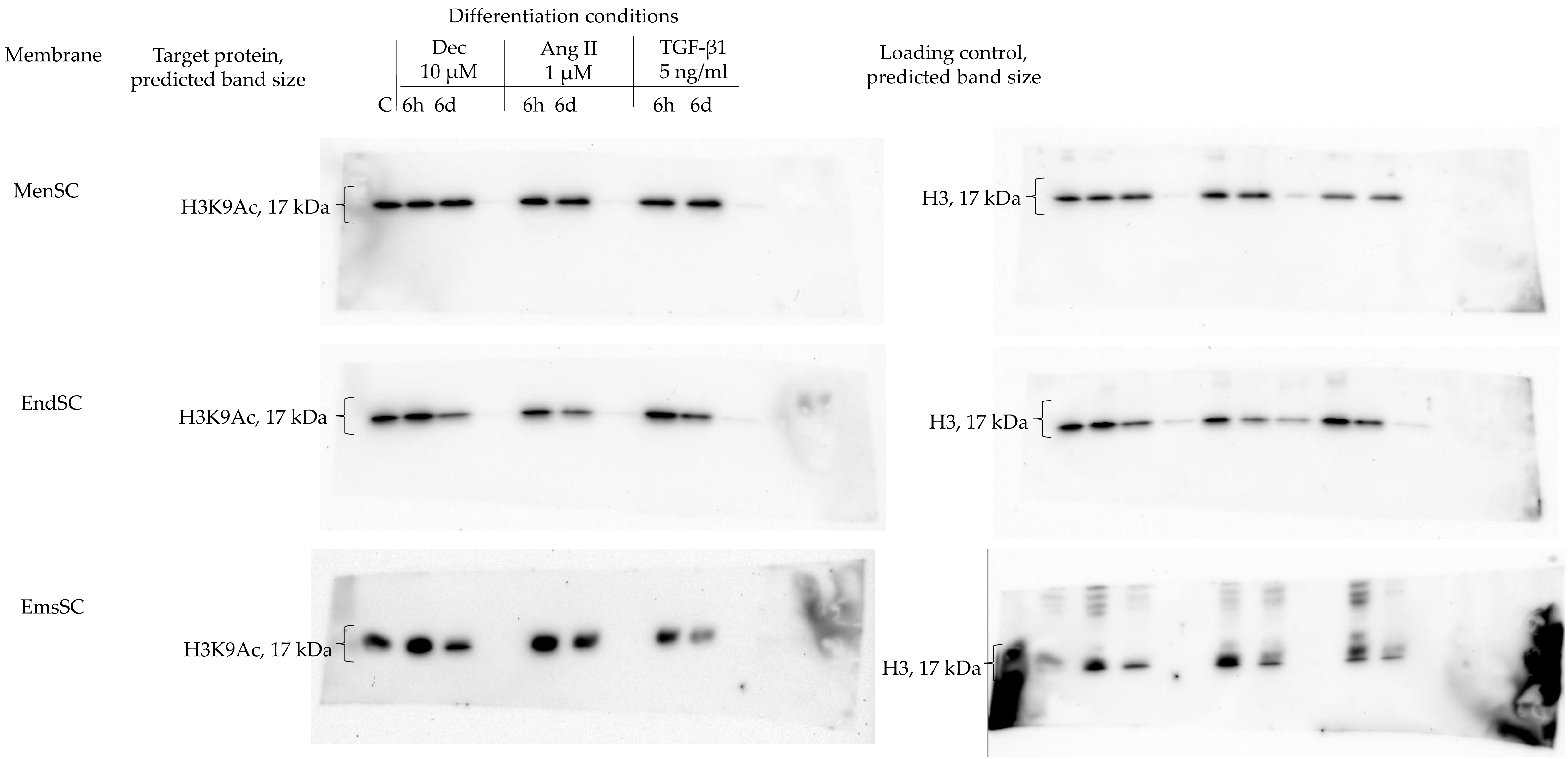

Supplementary figure 2. Western blot analysis of levels of H3K9Ac

Supplementary Table S1. PCR primers sequence for cDNA.

Name	Primer sequence	Product size, bp
GAPDH	F: GTGAACCATGAGAAGTATGACAAC<br>R: CATGAGTCCTTCCACGATACC	123
MESP1	F: TCGAAGTGGTTCCTTGGCAGAC<br>R: CCTCCTGCTTGCCTCAAAGTGTC	163
KDR	F: ACTGCAGTGATTGCCATGTTCT<br>R: CCTTCATTGGCCCGCTTAA	74
ISL1	F: GCGGAGTGTAATCAGTATTTGGA<br>R: GCATTTGATCCCGTACAACCT	102
A-Actinin	F: CGAGCGCCATGAACCAGATA<br>R: GTGGAACCGCATTTTTCCCC	293
cTNI	F: CCCTGCACCAGCCCCAATCAGT<br>R: CGAAGCCCAGCCCGGTCAACT	233
cTNT	F: CATGGAGAAGGACCTGAATGA<br>R: CGTCTCTCGATCCTGTCTTTG	108
NKX-2.5	F: CAAGTGTGCGTCTGCCTTT<br>R: GCGCACAGCTCTTTCTTTTC	105
MEF2C	F: GCCCTGAGTCTGAGGACAAG<br>R: AGTGAGCTGACAGGGTTGCT	163
Desmin	F: CCTACTCTGCCCTCAACTTC<br>R: AGTATCCCAACACCCTGCTC	519
Calponin	F: TCCAAATATGACCCCCAGAA<br>R: CCCACTCTCAAACAGGTCGT	276
KCND3	F: AGAGAGCTGATAAACGCAGGG<br>R: CAGGCAGTGCAGCAGGTGAT	209
CACNA1D	F: GGGCAATGGGACCTCATAAATAA<br>R: TTACCTGGTTGCGAGTGCATTA	141
HCN2	F: CGCCTGATCCGCTACATCCAT<br>R: AGTGCGAAGGAGTACAGTTCACT	230
SCN5A	F: TCATCGTAGACGTCTCTCTGGT<br>R: GGCTCTTGTTGTTCACGATGGT	318
KCNJ12	F: GCCAGCTAGGCTCTGTTTGTG<br>R: CTGAGACACATCTCTAAGGTAC	152
PDGFB	F: CTCGATCCGCTCCTTTGATGA<br>R: CGTTGGTGCGGTCTATGAG	239
TGFBR1	F: CACAGAGTGGGAACAAAAAGGT<br>R: CCAATGGAACATCGTCGAGCA	143
VEGFA	F: AGGGCAGAATCATCACGAAGT<br>R: AGGGTCTCGATTGGATGGCA	75
FOXO1	F: TCGTCATAATCTGTCCCTACACA<br>R: CGGCTTCGGCTCTTAGCAAA	168
mTOR	F: TTGAGGTTGCTATGACCCGAGAGAA<br>R: TTACCAGAAAGGGCACCAGCCAATA	566
NOTCH1	F: GGTGAGACCTGCCTGAATG<br>R: GTTGGGGTCCTGGCATC	102
WNT4	F: AGGAGGAGACGTGCGAGAAA<br>R: CGAGTCCATGACTTCCAGGT	83

Supplementary Table S2. PCR primers sequence for genomic DNA.

Name	Primer sequence	Product size, bp
GAPDH promoter	F: TACTAGCGGTTTTACGGGCG<br>R: GGCTGCGGGCTCAATTTATAG	135
WNT4 promoter	F: TCCTCCCAATCACAGCGTCT<br>R: GTGGGAATCCGAAACCTCGC	127
FOXO1 promoter	F: GCTCTGCTGCTCCGTAGTAA<br>R: TCTCTCGCCTTCTCAGTGTT	88
RARβ promoter	F: GTTGGGTCATTTGAAGGTTAGCAG<br>R: ACAAACCCTGCTCGGATCGCT	223
EED	F: GCGCTTTGAAATCCACCCTG<br>R: GGCGAATGGAAAGTACCCGT	168
EZH2	F: CGCCGGTTCCCGCCAAGAG<br>R: GTTCGCTGTAAGGGACGCCA	226
